# NEAT1 aggravates sepsis-induced acute kidney injury by sponging miR-22-3p

**DOI:** 10.1515/med-2020-0401

**Published:** 2020-04-20

**Authors:** Yawei Feng, Jun Liu, Ranliang Wu, Peng Yang, Zhiqiang Ye, Furong Song

**Affiliations:** Department of Anesthesiology, The Third Affiliated Hospital, Sun Yat-sen University, Guangzhou, China; Department of Anesthesiology, The First Affiliated Hospital, Sun Yat-sen University, Guangzhou, Guangdong, No. 58, Zhongshan Erlu, Guangzhou 510080, China; Department of Emergency, The Third Affiliated Hospital, Sun Yat-Sen University, Guangzhou, China

**Keywords:** sepsis, NEAT1, miR-22-3p, acute kidney injury, NF-κB pathway

## Abstract

**Background and aim:**

Acute kidney injury (AKI) is a common complication of sepsis. Long noncoding RNA nuclear-enriched abundant transcript 1 (NEAT1) plays a vital role in various diseases, including AKI. This study aimed to investigate the function and mechanism of NEAT1 in sepsis-induced AKI.

**Materials and methods:**

A septic AKI model was established by treating HK-2 cells with lipopolysaccharide (LPS). The levels of NEAT1 and miR-22-3p were measured by quantitative real-time PCR. Cell apoptosis was assessed by flow cytometry. The levels of apoptosis-related protein and autophagy-related factors were examined by the western blot assay. An enzyme-linked immunosorbent assay was used to calculate the contents of inflammatory factors. The interaction between NEAT1 and miR-22-3p was validated by dual-luciferase reporter assay, RNA immunoprecipitation assay, and RNA pull-down assay. The levels of nuclear factor (NF)-κB pathway-related proteins were evaluated by the western blot assay.

**Results:**

NEAT1 was upregulated, while miR-22-3p was downregulated in patients with sepsis and in LPS-stimulated HK-2 cells. LPS treatment triggered cell apoptosis, autophagy, and inflammatory response in HK-2 cells. NEAT1 knockdown attenuated LPS-induced cell injury. NEAT1 modulated LPS-triggered cell injury by targeting miR-22-3p. Furthermore, NEAT1 regulated the NF-κB pathway by modulating miR-22-3p.

**Conclusion:**

Depletion of NEAT1 alleviated sepsis-induced AKI via regulating the miR-22-3p/NF-κB pathway.

## 1 Introduction

Sepsis is defined as a syndrome of the dysregulated host response to infection, resulting in life-threatening organ dysfunction [1]. Sepsis is one of the major causes of the global burden of diseases, leading to approximately 8 million deaths every year [2]. Acute kidney injury (AKI) has been recognized as a fatal complication of the illness; more than 50% of AKI are caused by sepsis [3]. AKI caused by sepsis is closely related to the high mortality of up to 60–80% [4]. Although numerous studies have investigated the pathogenesis of sepsis-induced AKI, its pathophysiological mechanisms are still complex and have not been fully understood [5]. Hence, it is imperative to seek novel biomarkers for sepsis-induced AKI.

Long noncoding RNAs (lncRNAs) are a type of ncRNAs, consisting of more than 200 nucleotides [6]. In recent years, accumulating evidence has suggested that lncRNAs are relevant regulators in a variety of biological processes, including innate immunity and inflammatory responses [7,8]. LncRNA nuclear-enriched abundant transcript 1 (NEAT1) has been reported to play a crucial role in the innate immune response. Previous research has corroborated that NEAT1 aggravated ischemia-induced AKI via directly targeting miR-27a-3p [9]. Moreover, NEAT1 promoted sepsis-induced brain injury in mice by mediating the nuclear factor (NF)-κB pathway [10]. However, the mechanism of NEAT1 in sepsis-induced AKI remains poorly investigated.

MicroRNAs (miRNAs) are highly conserved short ncRNAs, consisting of 18–25 nucleotides [11]. miRNAs are post-transcriptional modulators that involve in various pathological processes of disorders [12]. miRNAs are critical regulators of the inflammatory response [13]. Recent studies have suggested that lncRNAs serve as competing endogenous RNAs (ceRNAs) to downregulate miRNA expression [14]. Previous research showed that the miR-22-3p expression was reduced in patients with sepsis [15]. However, the molecular mechanism of miR-22-3p in sepsis-induced AKI remains unknown.

In this study, the level of NEAT1 in the serum of the patients with sepsis-induced AKI was detected. We established a septic AKI cell model by inducing lipopolysaccharide (LPS) in HK-2 cells. Then, we explored the function and molecular mechanism of NEAT1 in sepsis-induced AKI.

## 2 Materials and methods

### 2.1 Serum samples

Eighteen patients with sepsis-induced AKI and 18 healthy volunteers as normal controls were recruited from The Third Affiliated Hospital, Sun Yat-sen University. All participants signed written informed consents. This study was approved by the Ethics Committee of The Third Affiliated Hospital, Sun Yat-sen University. All serum specimens were centrifuged at 8,000 × *g* for 3 min.

### 2.2 Cell culture

Human renal tubular epithelial cell line (HK-2) was purchased from Y-J Biological (Shanghai, China). The cells were cultured in RPMI-1640 medium (Gibco, Carlsbad, CA, USA) supplemented with 10% fetal bovine serum (Gibco). All cells were maintained at 37°C with 5% CO_2_. To establish a septic AKI cell model, HK-2 cells were treated with 1.0 μg/mL LPS (Sigma-Aldrich, St Louis, MO, USA) for 24 h.

### 2.3 Plasmids and cell transfection

Small interference RNA (siRNA) for NEAT1 (si-NEAT1) and the control siRNA (si-NC), miR-22-3p mimic (miR-22-3p) and the control mimic (NC), miR-22-3p inhibitor (anti-miR-22-3p) and the control inhibitor (anti-NC), NEAT1 overexpression vector (pcDNA-NEAT1), and the empty vector (pcDNA-NC) were purchased from RiboBio (Guangzhou, China). All plasmids and oligonucleotides were transfected into an LPS-induced septic AKI model using Lipofectamine 2000 reagent (Invitrogen, Carlsbad, CA, USA).

### 2.4 Quantitative real-time PCR

Total RNA was isolated from serum samples or cells using miRNeasy Serum/Plasma Kit (Qiagen, Hilden, Germany) or TRIzol reagent (Invitrogen). Total RNA was reversely transcribed into cDNA using the Reverse Transcription System (Promega, Madison, WI, USA) or miScript RT Kit (Qiagen). PCR amplification was performed using SYBR Green PCR Master Mix (Thermo Fisher Scientific, Waltham, MA, USA). The relative expression of NEAT1 and miR-22-3p was calculated by the 2^−ΔΔCt^ method. Glyceraldehyde-3-phosphate dehydrogenase (GAPDH) and U6 were used as endogenous controls. All primers were synthesized in Sangon Biotech (Shanghai, China) and are as follows: NEAT1 forward, 5′-TGGCTAGCTCAGGGCTTCAG-3′, reverse, 5′-TCTCCTTGCCAAGCTTCCTTC-3′; miR-22-3p forward, 5′-AAGCTGCCAGTTGAAGAACTGTA-3′, reverse, 5′-GCTGTCAACGATACGCTACGTAAC-3′; GAPDH forward, 5′-TGCACCACCAACTGCTTAGC-3′, reverse, 5′-GGCATGGACTGTGGTCATGAG-3′; U6 forward, 5′-CTCGCTTCGGCAGCACA-3′, reverse, 5′-AACGCTTCACGAATTTGCGT-3′.

### 2.5 Flow cytometry

Cell apoptosis was detected using Annexin V, FITC Apoptosis Detection Kit (Dojindo, Kumamoto, Japan). Briefly, the HK-2 cells were resuspended and seeded in 96-well plates. Then, the cells were stained with FITC-Annexin V and propidium iodide. The cell apoptotic rate was monitored by flow cytometry (BD Biosciences, San Jose, CA, USA).

### 2.6 Western blot assay

Total protein was extracted with RIPA buffer (Thermo Fisher Scientific). Protein concentration was detected using the Easy II Protein Quantitative Kit (BCA) (TransGen Biotech, Beijing, China). Then, the proteins were separated by sodium dodecyl sulfate-polyacrylamide gel electrophoresis and transferred to polyvinylidene fluoride membranes (Millipore, Billerica, MA, USA). The membranes were incubated at 4°C overnight with primary antibodies against Bax (Abcam, Cambridge, UK), B-cell lymphoma 2 (Bcl-2; Abcam), cleaved caspase 3 (Abcam), Beclin-1 (Abcam), microtubule-associated protein 1 light chain 3 (LC3)-I (Abcam), LC3-II (Abcam), p-IκBα (Abcam), IκBα (Abcam), p-p65 (Abcam), p65 (Abcam), or GAPDH (Abcam) at a dilution ratio of 1:1,000. Next, the membranes were incubated with the corresponding horseradish peroxidase–conjugated secondary antibody (1:4,000; Abcam) for 2 h at room temperature. The protein bands were visualized by enhanced chemiluminescence reagents (Millipore).

### 2.7 Enzyme-linked immunosorbent assay

The contents of tumor necrosis factor-alpha (TNF-α), interleukin 6 (IL-6), interleukin 8 (IL-8), and interleukin 1 beta (IL-1β) were detected using the enzyme-linked immunosorbent assay (ELISA) kit (RD, Minneapolis, MN, USA). The concentration was quantified in pg/mL.

### 2.8 Dual luciferase reporter assay

The fragment of NEAT1 harboring miR-22-3p binding sites and the mutant was cloned into pGL3 plasmid (Promega) to obtain pGL3-NEAT1-wt and pGL3-NEAT1-mut vectors, respectively. Then, the cells were cotransfected with miR-22-3p mimic (miR-22-3p) or the control mimic (NC) and pGL3-NEAT1-wt or pGL3-NEAT1-mut. Luciferase intensity was assessed using the Dual-Luciferase Reporter Assay System (Promega).

### 2.9 RNA immunoprecipitation assay

RNA immunoprecipitation (RIP) assay was performed with EZ-Magna RIP Kit (Millipore) according to the manufacturer’s instructions. First, the transfected cells were lysed using RIP lysis buffer. The cell lysates were collected and incubated with magnetic beads conjugated with anti-Ago2 or the control anti-IgG for 4 h at 4°C. Then, the expression of immunoprecipitate was detected by quantitative real-time PCR (qRT-PCR).

### 2.10 RNA pull-down assay

The biotin-labeled miR-22-3p probe (Bio miR-22-3p) and the negative control probe (Bio-NC) were purchased from RiboBio. Briefly, the biotinylated probe was incubated with M-280 Streptavidin Dynabeads (Invitrogen) at 37°C for 2 h to construct probe-coated beads. Then, the cells were lysed and incubated with probe-coated beads at 4°C for 3 h. After RNA isolation, the enrichment of NEAT1 was examined by qRT-PCR.

### 2.11 Statistical analyses

All data were presented as mean ± standard deviation. Statistical analyses were performed using GraphPad Prism 7.0 software (GraphPad, San Diego, CA, USA). Student’s *t*-test or one-way analysis of variance was performed to analyze the differences. When the *P* value is <0.05, the difference was considered statistically significant. All experiments were performed at least three times independently.

## 3 Results

### 3.1 LncRNA NEAT1 was upregulated and miR-22-3p was downregulated in patients with sepsis and in LPS-induced HK-2 cells

**Figure 1:**
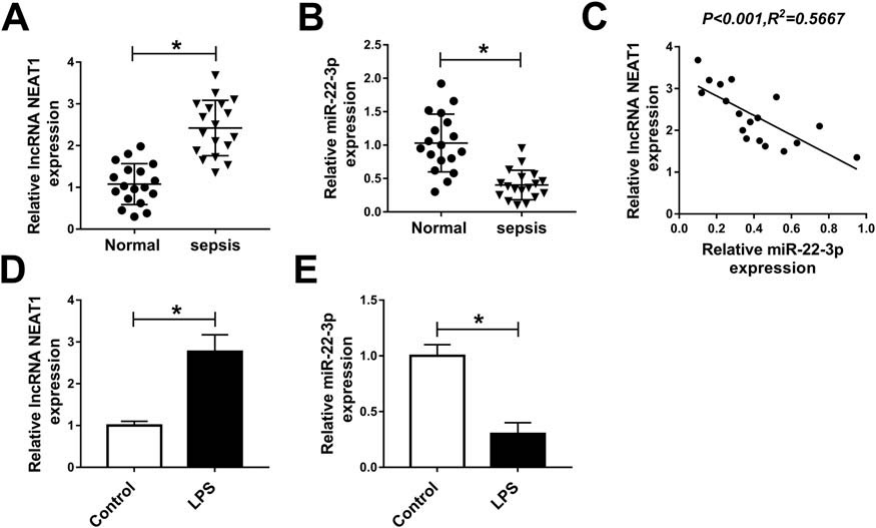
The expression of NEAT1 and miR-22-3p in patients with sepsis and in LPS-induced HK-2 cells. (A and B) The expression levels of NEAT1 and miR-22-3p were detected in the serum of patients with sepsis-induced AKI (*n* = 18) and healthy volunteers (*n* = 18). (C) NEAT1 expression was inversely correlated with miR-22-3p expression. (D and E) The expression levels of NEAT1 and miR-22-3p were measured in LPS-induced HK-2 cells and HK-2 cells (control) by qRT-PCR. *P < 0.05.

First, we examined the levels of NEAT1 and miR-22-3p in the serum of patients with sepsis and in healthy controls. The results revealed that NEAT1 expression was dramatically increased (Figure 1A), while the miR-22-3p expression was significantly decreased in patients with sepsis compared with healthy controls (Figure 1B). Moreover, the levels of NEAT1 and miR-22-3p were negatively correlated in patients with sepsis (Figure 1C). We also investigated the levels of NEAT1 and miR-22-3p in LPS-induced HK-2 cells. The results revealed that the NEAT1 expression was remarkably higher, whereas the miR-22-3p expression was markedly lower in LPS-induced HK-2 cells than in untreated cells (Figure 1D and E). These data evidenced that NEAT1 played an important role in sepsis-triggered AKI.

### 3.2 LPS treatment induced cell apoptosis, autophagy, and inflammatory response in HK-2 cells

**Figure 2:**
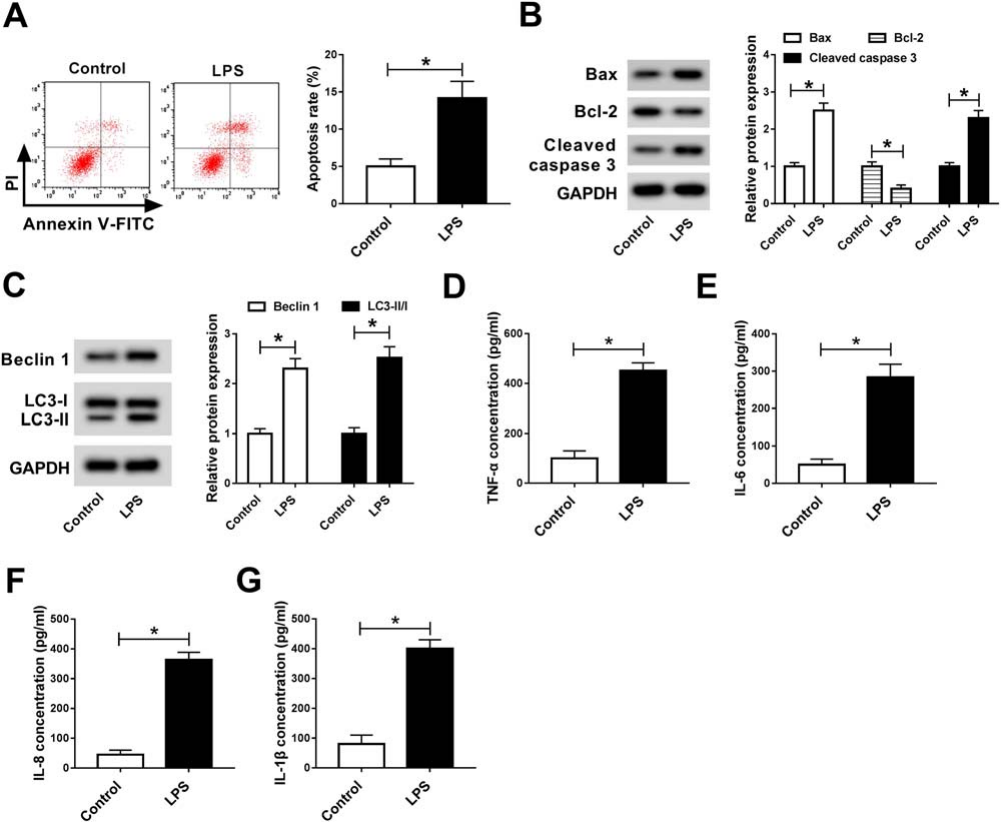
LPS treatment induced cell apoptosis, autophagy, and inflammatory response in HK-2 cells. (A) Cell apoptosis rate was examined by flow cytometry. (B) Western blot assay detected the levels of apoptosis-related proteins (Bax, Bcl-2, and cleaved caspase 3). (C) The levels of autophagy-related factors (Beclin-1 and LC3-II/I) were detected by western blot analysis. (D–G) The concentration of inflammatory factors (TNF-α, IL-6, IL-8, and IL-1β) was measured by ELISA assay. *P < 0.05.

To investigate the effect of LPS on cell injury, we detected cell apoptosis and inflammatory factor in LPS-stimulated HK-2 cells. Flow cytometry suggested that the cell apoptosis rate was remarkably increased in LPS-stimulated cells compared with that of untreated cells (Figure 2A). Consistently, LPS caused a dramatic decrease in Bcl-2 protein levels and an evident increase in Bax and cleaved caspase 3 protein levels (Figure 2B). Western blot assay revealed that autophagy-related factors (Beclin-1 and LC3-II/I) were obviously increased after treatment with LPS (Figure 2C). In addition, ELISA showed that the concentration of inflammatory cytokines (TNF-α, IL-6, IL-8, and IL-1β) was overtly increased in LPS-stimulated cells in comparison with untreated cells (Figure 2D–G). All these data indicated that LPS triggered cell apoptosis, autophagy, and inflammatory response in HK-2 cells.

### 3.3 NEAT1 knockdown attenuated LPS-induced injury in HK-2 cells

**Figure 3:**
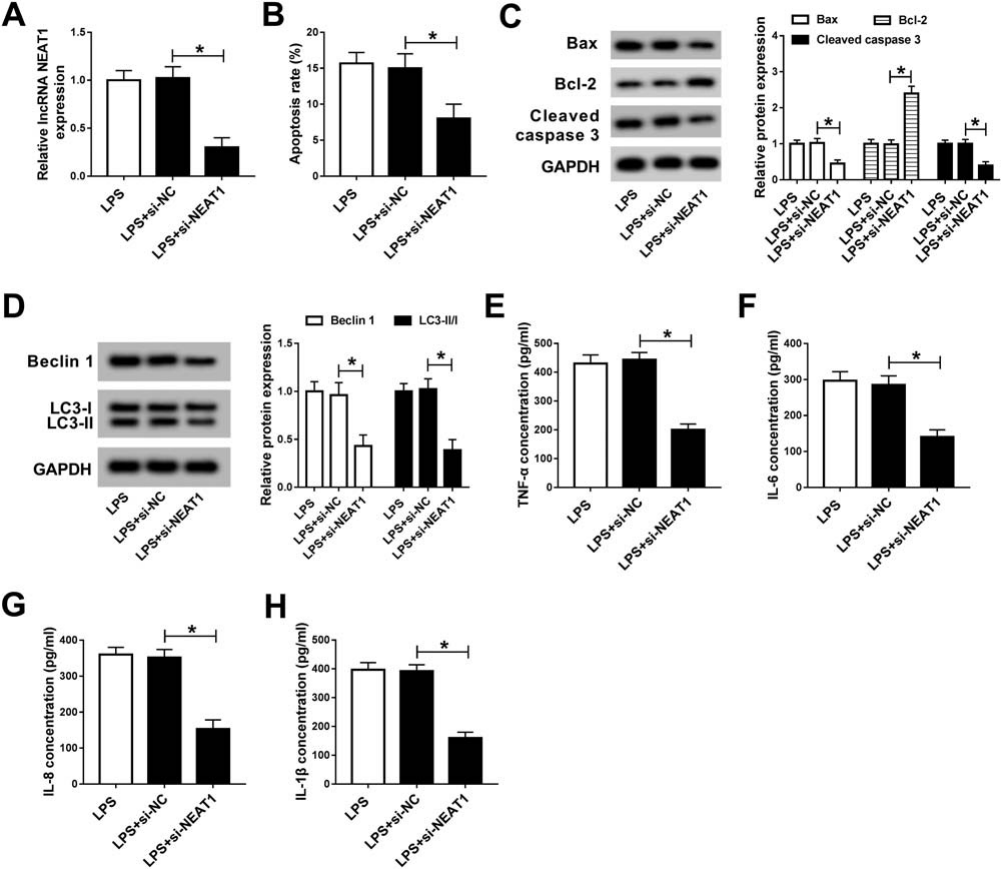
NEAT1 knockdown attenuated LPS-induced injury in HK-2 cells. (A–G) LPS-stimulated HK-2 cells were transfected with si-NC or si-NEAT1. (A) NEAT1 expression was detected by qRT-PCR. (B) Cell apoptosis rate was evaluated by flow cytometry. (C) The levels of apoptosis-related proteins (Bax, Bcl-2, and cleaved caspase 3) were examined using western blot. (D) The levels of autophagy factors were tested by the western blot assay. (E–H) ELISA was used to measure the contents of inflammatory factors. *P < 0.05.

To explore the effect of NEAT1 on LPS-induced injury, LPS-treated HK-2 cells were transfected with si-NC or si-NEAT1. The results of qRT-PCR showed that NEAT1 was effectively repressed in the si-NEAT1 group (Figure 3A). Transfection of LPS-stimulated HK-2 cells with si-NEAT1 resulted in an obvious decrease in cell apoptosis rate (Figure 3B). Meanwhile, treatment with LPS and introduction of si-NEAT1 led to a significant increase in the protein expression of Bcl-2 and a dramatic decrease in the protein expression of Bax and cleaved caspase 3 in comparison with the LPS + si-NC group (Figure 3C). Moreover, depletion of NEAT1 restrained autophagy after treatment with LPS and transfection with si-NEAT1 via reducing the levels of autophagy factors (Figure 3D). ELISA revealed that the concentration of inflammatory factors (TNF-α, IL-6, IL-8, and IL-1β) was apparently decreased in the LPS + si-NEAT1 group compared with that of the LPS + si-NC group (Figure 3E–H). All these data corroborated that NEAT1 knockdown alleviated LPS-triggered cell injury in HK-2 cells.

### 3.4 NEAT1 directly targeted miR-22-3p

**Figure 4:**
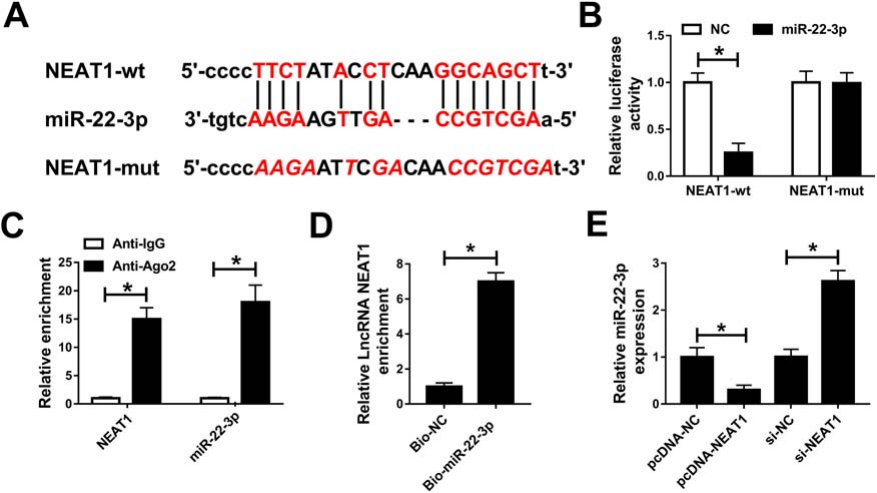
NEAT1 directly targeted miR-22-3p. (A) The putative binding sites of NEAT1 and miR-22-3p are shown. (B) Luciferase activity was detected in HK-2 cells cotransfected with pGL3-NEAT1-wt or pGL3-NEAT1-mut and miR-22-3p or NC. (C) RIP assay was performed to examine the enrichment of NEAT1 and miR-22-3p in the immunoprecipitated complex. (D) The interaction between NEAT1 and miR-22-3p was validated by the RNA pull-down assay. (E) The level of miR-22-3p was measured in HK-2 cells transfected with pcDNA-NC, pcDNA-NEAT1, si-NC, or si-NEAT1 using qRT-PCR. *P < 0.05.

To investigate the mechanism of NEAT1, we predicted that miR-22-3p was a putative target of NEAT1 by starBase v2.0 (Figure 4A). To further verify that NEAT1 targeted miR-22-3p, the dual-luciferase reporter assay was performed. The results showed that miR-22-3p evidently reduced the luciferase activity in HK-2 cells transfected with NEAT1-wt (Figure 4B). Furthermore, the RIP assay validated that both NEAT1 and miR-22-3p were enriched in the Ago2 antibody complex (Figure 4C). RNA pull-down assay indicated that NEAT1 was significantly enriched by Bio-miR-22-3p but not by Bio-NC (Figure 4D). In the meantime, the expression of miR-22-3p was measured in HK-2 cells transfected with pcDNA-NC, pcDNA-NEAT1, si-NC, or si-NEAT1. The qRT-PCR results revealed that overexpression of NEAT1 markedly reduced the miR-22-3p level, while silencing of NEAT1 strikingly increased the miR-22-3p level (Figure 4E). These results demonstrated that NEAT1 negatively regulated miR-22-3p in HK-2 cells.

### 3.5 Inhibition of miR-22-3p reversed the effect of NEAT1 knockdown on LPS-induced cell injury

**Figure 5:**
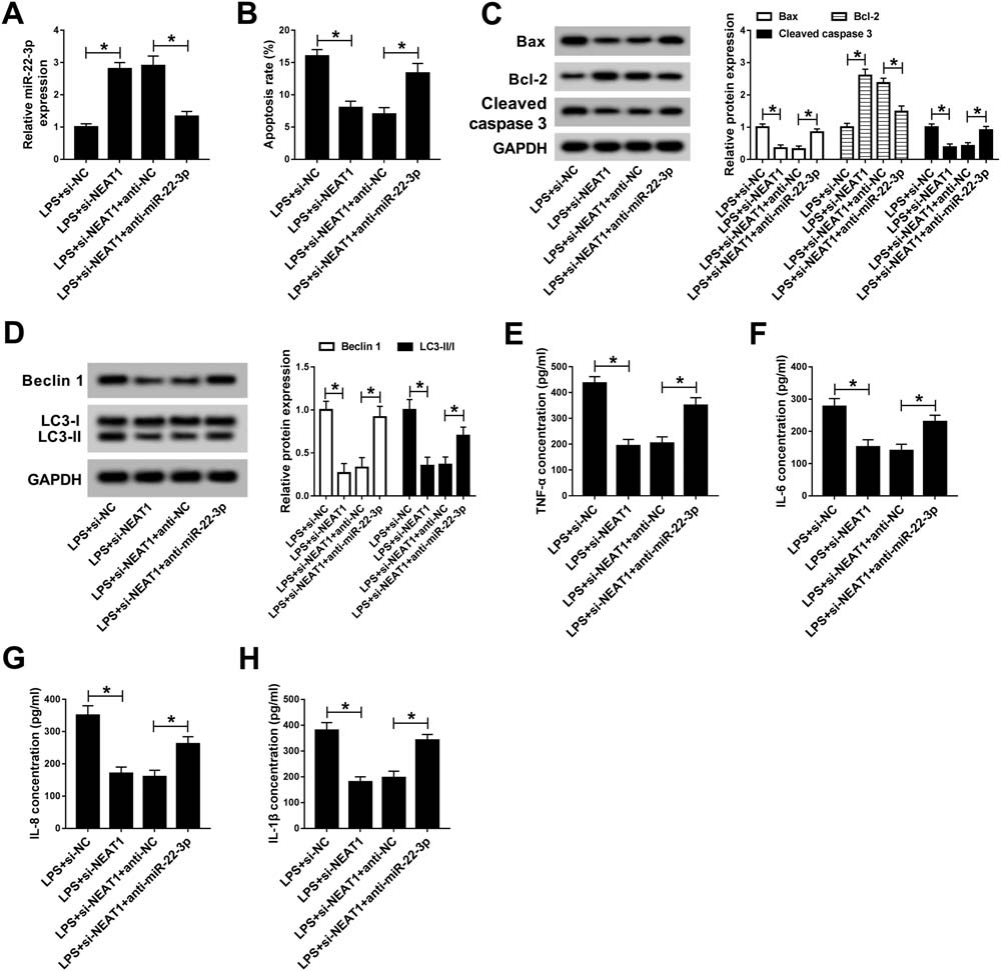
Inhibition of miR-22-3p reversed the effect of NEAT1 depletion on LPS-induced cell injury. (A–G) LPS-induced HK-2 cells were transfected with si-NC, si-NEAT1, si-NEAT1 + anti-NC, or si-NEAT1 + anti-miR-22-3p. (A) The expression of miR-22-3p was examined by qRT-PCR. (B) Cell apoptosis rate was evaluated by flow cytometry. (C) The levels of apoptosis-related proteins were measured by western blot. (D) The levels of autophagy factors were tested by the western blot assay. (E–H) ELISA was used to detect the contents of inflammatory factors. *P < 0.05.

To investigate the role of miR-22-3p in sepsis-induced AKI, LPS-treated HK-2 cells were transfected with si-NC, si-NEAT1, si-NEAT1 + anti-NC, or si-NEAT1 + anti-miR-22-3p. First, the qRT-PCR results showed that the miR-22-3p expression in the si-NEAT1 group was obviously higher than that in the si-NC group, while inhibition of miR-22-3p restored the effect triggered by NEAT1 depletion (Figure 5A). Flow cytometry assay revealed that NEAT1 silencing markedly decreased the cell apoptosis rate, while the effect was reversed by inhibiting NEAT1 and miR-22-3p (Figure 5B). In addition, the Bcl-2 expression was notably reduced, while Bax and cleaved caspase 3 expression was strikingly enhanced in the si-NEAT1 + anti-miR-22-3p group compared with that of the si-NEAT1 + anti-NC group (Figure 5C). Transfection with anti-miR-22-3p reversed the decrease in the levels of autophagy factors induced by NEAT1 knockdown (Figure 5D). ELISA indicated that si-NEAT1 markedly reduced the concentration of inflammatory factors (TNF-α, IL-6, IL-8, and IL-1β), which were stopped by inhibition of miR-22-3p (Figure 5E–H). These rescue experiments manifested that knockdown of miR-22-3p reversed the effect of NEAT1 inhibition on LPS-triggered injury in HK-2 cells.

### 3.6 NEAT1 regulated the NF-κB pathway in LPS-treated HK-2 cells

**Figure 6:**
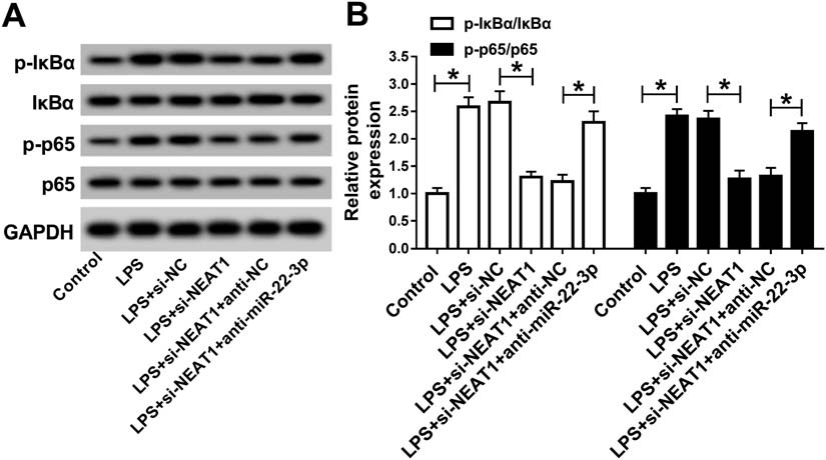
NEAT1 regulated the NF-κB pathway in LPS-induced HK-2 cells. (A and B) HK-2 cells were treated with LPS before the introduction of si-NC, si-NEAT1, si-NEAT1 + anti-NC, or si-NEAT1 + anti-miR-22-3p, and the phosphorylation of IκBα and p65 was tested by western blot analysis. *P < 0.05.

To explore whether the NF-κB pathway was activated in sepsis-induced AKI, we selected NF-κB pathway-related proteins (IκBα and p65) as surrogate markers. Western blot results showed that LPS treatment observably facilitated the phosphorylation of IκBα and p65 (Figure 6A and B), indicating that the NF-κB signal was activated. In addition, NEAT1 knockdown markedly hindered phosphorylation of IκBα and p65, which was reversed after transfection with the miR-22-3p inhibitor (Figure 6A and B). These results reflected that NEAT1 knockdown inhibited the activation of the NF-κB pathway through modulation of miR-22-3p in LPS-treated HK-2 cells.

## 4 Discussion

Pathophysiological mechanisms of sepsis-induced AKI include microvascular dysfunction and inflammation [16]. The leading causes of AKI are sepsis, acute ischemia, and hypoxia [17]. Increasing evidence has elucidated that lncRNA NEAT1 is involved in mediating innate immunity. Chen *et al.* revealed that NEAT1 knockdown inhibited the immunity in mice with sepsis through sponging miR-125 and downregulating MCEMP1 [18]. A previous study suggested that lncRNA NEAT1 contributed to sepsis-triggered AKI via repressing miR-204 and activating the NF-κB pathway [19]. Besides, Jiang *et al*. revealed that NEAT1 aggravated ischemia-induced injury via functioning as a ceRNA of miR-27a-3p [9]. Similar to previous research studies, the NEAT1 expression was dramatically increased in patients with sepsis in comparison with healthy controls. In addition, knockdown of NEAT1 alleviated LPS-triggered injury in HK-2 cells, indicating that NEAT1 might play a role in sepsis-triggered AKI progression.

The pathophysiological mechanisms of sepsis-triggered AKI are complex, including the inflammatory immune response triggered by mitochondrial damage and oxidative stress [20]. The previous study has demonstrated that apoptosis and autophagy provided potential therapeutic targets for AKI [21]. Additionally, it has been proved that inflammation and cell apoptosis are crucial mechanisms of AKI [22]. Consistent with previous studies, we verified that inhibition of NEAT1 alleviated sepsis-stimulated AKI by inhibiting the intrinsic apoptosis, autophagy, and inflammatory response.

In this study, we determined that NEAT1 was a sponge of miR-22-3p and negatively regulated miR-22-3p. Interestingly, increasing evidence has elucidated that lncRNAs function as ceRNAs through sponging miRNAs [23]. A previous study has demonstrated that miR-22-3p was downregulated in patients with sepsis [15]. Consistent with the previous research, the miR-22-3p expression was decreased in patients with sepsis and in LPS-induced HK-2 cells. Next, the rescue experiments validated the hypothesis that NEAT1 regulated LPS-induced cell injury by acting as a sponge for miR-22-3p.

Accumulating evidence has suggested that the NF-κB pathway plays a vital role in sepsis-stimulated AKI [24,25,26]. Li *et al.* found that TIMP2 ameliorated sepsis-triggered AKI by regulating the NF-κB pathway [27]. Glycyrrhizic acid alleviated sepsis-induced AKI through inhibiting the activation of the NF-κB signaling pathway [28]. In this study, the phosphorylation of IκBα and p65 was decreased by knockdown of NEAT1, which was reversed by suppression of miR-22-3p. From these data, we confirmed that NEAT1 aggravated LPS-induced cell injury via activating the NF-κB pathway.

In conclusion, the study demonstrated that NEAT1 was upregulated, while miR-22-3p was downregulated in patients with sepsis and in LPS-induced HK-2 cells. NEAT1 knockdown could attenuate LPS-induced cell injury via increasing the miR-22-3p expression and inhibiting the NF-κB pathway, indicating that NEAT1 might be a biomarker for sepsis-induced AKI.
